# Investigating the effect of telmisartan on acrylamide-induced neurotoxicity through *in vitro* and *in vivo* methods

**DOI:** 10.22038/IJBMS.2023.69636.15167

**Published:** 2023

**Authors:** Zahra Yazdanpanah, Mahboobeh Ghasemzadeh Rahbardar, Bibi Marjan Razavi, Hossein Hosseinzadeh

**Affiliations:** 1 Department of Pharmacodynamics and Toxicology, School of Pharmacy, Mashhad University of Medical Sciences, Mashhad, Iran; 2 Pharmaceutical Research Center, Pharmaceutical Technology Institute, Mashhad University of Medical Sciences, Mashhad, Iran; 3 Targeted Drug Delivery Research Center, Pharmaceutical Technology Institute, Mashhad University of Medical Sciences, Mashhad, Iran

**Keywords:** Anti-oxidants, Cell survival, Motor disorders, Neurotoxins, PC12 cells, Vitamin E

## Abstract

**Objective(s):**

Acrylamide (ACR) is an environmental contaminant and neurotoxin. Telmisartan is an AT1 blocker that has neuroprotective properties basically through its anti-oxidant effect. The effect of telmisartan on ACR-induced neurotoxicity was investigated in this study.

**Materials and Methods:**

Male Wistar rats were randomly assigned to eight groups (n=6): 1:Control (normal saline), 2:ACR (50 mg/kg, 11 days, IP), 3:ACR+vitamin E (200 mg/kg, every other day, 11 days), 4-6:ACR+telmisartan (0.6, 1.25, and 2.5 mg/kg, 11 days, IP), 7:ACR+telmisartan (0.6 mg/kg, days 3–11), 8:Telmisartan (2.5 mg/kg, 11 days). The behavioral test and blood pressure were assessed after 11 days. Then, the levels of MDA and GSH in brain tissue were measured. The MTT assay was used to evaluate the effect of telmisartan on ACR-induced cytotoxicity.

**Results:**

Exposing PC12 cells to ACR decreased cell viability versus the control group. Pretreating PC12 cells with telmisartan (0.0125, 0.025 µM) enhanced cell viability compared with the ACR group. Compared with control samples, ACR significantly caused motor impairment, elevated MDA, and reduced GSH levels. Locomotor abnormalities were significantly ameliorated by telmisartan (0.6, 1.25 mg/kg, 11 days) and vitamin E versus the ACR group. Receiving telmisartan (0.6, 1.25, and 2.5 mg/kg) and vitamin E along with ACR decreased MDA levels and enhanced GSH content compared with the ACR group. There was no significant difference in animal blood pressure between the groups.

**Conclusion:**

Oxidative stress has a chief role in the neurotoxicity of ACR. Telmisartan (in doses that do not affect blood pressure) ameliorated ACR-induced toxicity by inhibiting oxidative stress.

## Introduction

A water-soluble alkene called acrylamide (ACR) is largely used to make polyacrylamide, which is utilized in personal care products, as well as in several chemical industries, wastewater treatment procedures, chemical grouting, and soil conditioning (1, 2). Plant-based meals including potatoes, grain goods, and roasted coffee contain ACR (2). The ACR content of 43,419 food products was the subject of a survey report published in 2015 by the European Food Safety Authority (EFSA). The findings revealed that whereas coffee had the greatest ACR concentration at 4.5 mg/kg, the average ACR content in fried potato products was as high as 1.0 mg/kg (3). The European Commission also set the limits for ACR residue in potato chips at 750 µg/kg and roast coffee and coffee compounds at 400–850 µg/kg (4). ACR monomeric form is very toxic (5) and has carcinogenic (6), teratogenic (7), neurotoxic, and hepatotoxic (8) effects. Its polymeric form is non-toxic (1). 

ACR causes neurotoxicity in humans, which manifests as skeletal muscle weakness, lethargy, weight loss, gait abnormalities, numbness of the extremities, ataxia, and polyneuropathy (9, 10). It has been proposed that one of the mechanisms of neurotoxicity brought on by ACR is oxidative stress. Oxidative damage is caused by an inequality between the overproduction of reactive oxygen species (ROS) and the elimination of free radicals. Excessive free radical generation caused by oxidative metabolism imbalance causes a cascade of changes such as mitochondrial dysfunction, protein and deoxyribonucleic acid (DNA) damage, inflammation, and energy deficiency (11). According to some *in vivo* (on rodents) and *in vitro* (on PC12 cells) studies, ACR can cause an imbalance in nervous tissue by increasing lipid peroxidation and decreasing anti-oxidant capacity, as evidenced by increased levels of MDA and ROS, as well as decreased levels of catalase, glutathione (GSH), superoxide dismutase (SOD), and glutathione peroxidase (GSH-Px) (11-13).

The abundance of ACR in food products and the environment necessitates immediate attention to its toxicity and risk to human health. Therefore, it appears that research into compounds that can inhibit ACR-induced toxicity is critical.

Telmisartan, an antihypertensive drug, is a highly lipid-soluble angiotensin II type 1 receptor blocker. This medicine demonstrated an anti-oxidant effect in several neurodegenerative disorders including multiple sclerosis and Huntington’s disease by enhancing GSH amount and decreasing MDA level (14, 15).

Hence, in the current study, the PC12 cells were used in the *in vitro* study, and the MTT test was performed to examine the effects of telmisartan on the prevention of ACR toxicity in these cells. Moreover, our team focused on assessing behavioral factors (gait score) and oxidative stress factors (MDA, GSH) in brain tissue to determine the probable protective effects of telmisartan against ACR-induced neurotoxicity in Wistar rats. 

## Materials and Methods

***Chemicals***

KH_2_PO_4_, KCl, Na_2_HPO_4_, ACR, trichloroacetic acid (TCA), phosphoric acid, NaOH, thiobarbituric acid (TBA), n-butanol, and dimethyl sulfoxide (DMSO) were obtained from Merck, Germany. Telmisartan was obtained from Tinab Shimi, Iran. Vitamin E was acquired from Osveh Pharmaceutical Co., Iran. RPMI-1640 medium was bought from Bonyakhteh, Iran. Absolute ethanol was procured from Simin Tak Co., Iran. Trypsin–ethylenediamine tetraacetic acid (EDTA) solution, fetal bovine serum (FBS), and pen-strep were purchased from Gibco, USA. Trypan blue, tween 20%,3-(4, 5-dimethylthiazol-2-yl)-2, 5-diphenyl tetrazolium bromide (MTT), and 5, 5’-dithiobis-(2-nitrobenzoic acid) (DTNB) were bought from Sigma-Aldrich, Germany.

***Study design***

This study was carried out in two parts: *in vitro* (cell culture) and *in vivo* (animal study).

***In vitro experiments***

***PC12 cell line***

PC12 cells (derived from a rat adrenal tumor, which was a derivative of the adrenergic neural crest) were prepared from the Pasteur Institute, Tehran, Iran. In a humidified environment with 5% CO_2_ at 37 °C, PC12 cells were cultured in RPMI 1640 media supplemented with 10% (v/v) FBS, 100 U/ml penicillin, and 100 g/ml streptomycin, and passaged at 80% confluence (2).

***Cell viability assay***

The PC12 cell line was cultured in 96-well microplates at 4,000 cells per well. The cells were treated with ACR (1–20 mM) for 24 hr after they had adhered to the substrate and begun a logarithmic growth phase. After that, the MTT assay was used to determine the viability of the cells, and GraphPad Prism 8 statistical software was used to analyze dose-dependent prevention to determine the half-maximal inhibitory concentration (IC_50_) value for a 24-hour exposure to ACR. Then, PC12 cells were cultured in 96-well microplates, treated with telmisartan (0.0125–1.6 µM for 24 hr, and then exposed to ACR 6 µM (final concentration calculated based on IC_50_ concentration assay) to assess the effect of telmisartan on ACR cytotoxicity. The cells were exposed to MTT solution 0.5 mg/ml (final concentration) for 3 hr in an incubator. The purple formazan result was then dissolved in 100 μl of DMSO after removing the medium-upper level. Using a microplate reader (Start Fax-2100, UK), the absorbance was estimated at 545 nm (using 630 nm as a reference). Finally, as a proportion of the value in control cultures, cell viability was reported (12).

***In vivo experiments***

***Animals***

***Induction of neurotoxicity in animals***

To induce neurotoxicity in male rats, ACR (50 mg/kg) was administered intraperitoneally and daily for 11 days (16).

To investigate the protective effects of telmisartan in neurotoxicity induced by ACR, telmisartan was administered intraperitoneally with doses 0.6, 1.25, and 2.5 mg/kg. The doses of telmisartan were selected according to our pilot studies (these doses did not reduce blood pressure).

All the injections, except for the positive control group (vitamin E), which was administered every other day, were done daily and at a specific time.

The tested groups were examined as follows:

1: Control group: animals receiving vehicle (normal saline plus 20 µl of Tween 20%);

2: Animals receiving a dose of 50 mg/kg of ACR intraperitoneally for 11 days (8);

3: Animals receiving a dose of 50 mg/kg of ACR+ vitamin E with a dose of 200 mg/kg, every other day intraperitoneally (17); 

4–6: Pre-treatment groups: animals receiving a dose of 50 mg/kg of ACR+ telmisartan (0.6, 1.25, and 2.5 mg/kg intraperitoneally for 11 days;

7: Treatment group: animals receiving a dose of 50 mg/kg of ACR+ telmisartan with a dose of 0.6 mg/kg intraperitoneally from day 3 to 11;

8: Animals receiving telmisartan with a dose of 2.5 mg/kg alone.

The number of animals in each group was 6.

***Evaluation of gait score***

After the end of the administration period, the rats were placed in a box with dimensions of 100 cm x 100 cm, and their movements were examined for 3 min. The movements of rats were graded as follows:

1) Stepping and walking are normal.

2) Stepping and walking are partially affected, and partial weakness and laxity of the hind limbs are evident.

3) Stepping and walking are moderately affected, and moderate weakness and laxity of hind limbs are evident.

4) Stepping and walking are severely affected and paralysis of the lower limbs has occurred (18).

***Systolic blood pressure measurement***

The systolic blood pressure of a rat tail was measured using a non-invasive tail-cuff method. The rat’s tail was cleaned and warmed using a light-emitting diode (LED) light. By pressing the corresponding key on the blood pressure monitor (NIBP Controller), air enters the blood pressure cuff and, after a short time, the heart rate returns to normal (a few seconds). The systolic blood pressure is the point at which the heartbeat of the animal resumes and the pulse amplitude returns to normal. After measuring the systolic blood pressure five times, the mean blood pressure was used to calculate the final systolic blood pressure (19).

***Collecting tissue samples***

After performing the behavioral tests, the animals of each group were sacrificed and their brain cortex tissue was isolated. The samples were first frozen in liquid nitrogen and then kept at -80 °C until further evaluation.

***Determining the amount of MDA***

MDA is a marker of lipid peroxidation and its increase indicates lipid peroxidation. MDA reacts with TBA in an acidic medium and forms a pink complex that has an absorption maximum of 532 nm (20).

First, 150–180 mg of each tissue sample was separated and tissue homogenate 10% was prepared with 1.15% cold KCl. Then 0.5 ml of this homogenate was mixed with 3 ml of 1% phosphoric acid and 1 ml of 0.6% TBA solution. The tubes containing this mixture were placed in boiling water for 45 min. To extract the colored complex, 4 ml of n-butanol was added to the cooled mixture and vortexed for one minute. Then it was centrifuged at 3000 g for 10 min and after transferring the organic phase to new tubes, the absorbance was measured at the wavelength of 532 nm for each of the samples. A standard curve in the range of concentrations of 0–100 nmol/ml of MDA, and finally, the amount of MDA was reported in terms of nmol/g tissue (21, 22).

***Determining the amount of GSH***

The basis of this method is the reaction of free sulfhydryl groups with DTNB reagent in an alkaline environment. The resulting yellow complex has an absorption maximum of 412 nm (23).

First, 150–180 mg of each tissue sample was separated and prepared with 10% phosphate buffer (pH: 7.4). Then, the homogenized samples were mixed with 10% TCA at a ratio of 1:1 and centrifuged at 2500 g for 10 min after vortexing. Then the upper phase was separated and mixed with 2 ml of phosphate buffer (pH: 8). After adding 0.5 ml of 0.04% DTNB reagent, the absorbance at 412 nm wavelength was measured for each of the samples. To calculate the amount of GSH, a standard curve was drawn in the concentration range of 0–300 nmol/ml of GSH, and its amount was reported in terms of nmol/g tissue (19, 24).

***Statistical analysis***

Statistical calculations were performed using Graph Pad Prism 8 software program. The results are shown as Mean ± standard deviation (SD). One-way ANOVA and Tukey-Kramer posttest were used to compare different groups in the *in vitro* and *in vivo* experiments. Kruskal-Wallis non-parametric test and Dunn posttest were used to analyze the data of the behavioral test. These data are shown as Median ± interquartile range (IQR). *P*<0.05 was considered a significant difference.

## Results

***In vitro***

***Assessing the IC***_50_ ***of ACR***

**Figure 1:**
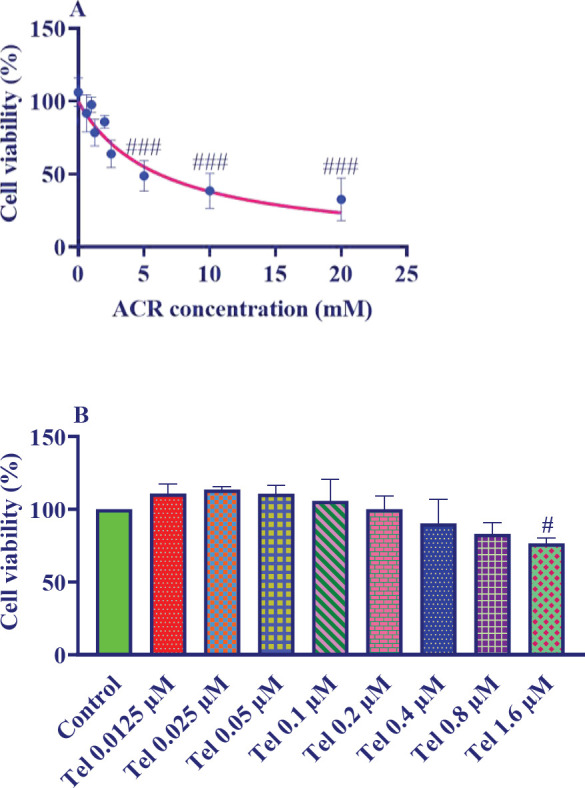
A: Effect of 1–20 mM ACR concentrations and B: 0–1.6 µM telmisartan on PC12 cells for 24 hr. The data are presented as mean±SD (n=4). ANOVA and post-test Tukey–Kramer were used for statistical analysis. ### *P*<0.001 and #* P*<0.05 compared with the control group

ACR: acrylamide; Tel: telmisartan

PC12 cells were exposed to different concentrations of ACR (0–20 mM) for 24 hr. The viability of these cells was measured using the MTT test. As shown in Figure 1A, the IC_50_ of ACR was 6 mM. 

***Effect of telmisartan on the viability of PC12 cells***

To investigate the effect of telmisartan on the viability of PC12 cells, these cells were exposed to different concentrations (0–1.6 µM) of telmisartan for 24 hr. Cell viability was tested using the MTT test. Telmisartan at concentrations higher than 0.8 μM significantly decreased the viability of PC12 cells (Figure 1B).

***Effect of telmisartan on the survival of PC12 cells exposed to ACR***

**Figure 2:**
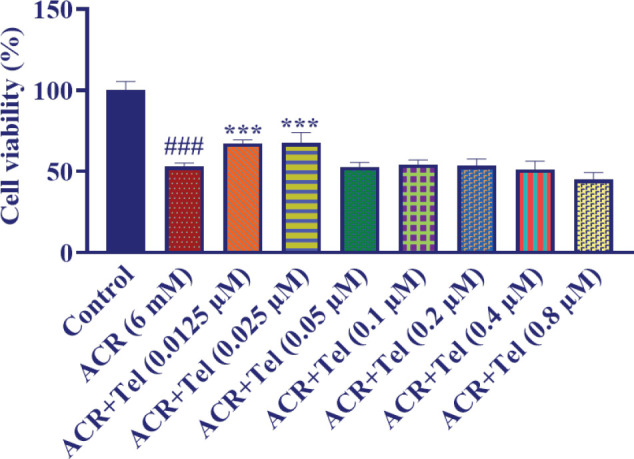
Effect of telmisartan (0.0125–0.8 µM) on the viability of PC12 cells exposed to ACR for 24 hr. The data are presented as mean±SD (n=4). ANOVA and post-test Tukey–Kramer were used for statistical analysis. ### *P*<0.001 compared with the control group, *** *P*<0.001 compared with the ACR group

The exposure to ACR (6 mM, 24 hr) significantly reduced the viability of PC12 cells compared with the control group (*P*<0.001). To examine the protective effect of telmisartan on the toxicity of ACR, first PC12 cells were treated with different concentrations of telmisartan (0–0.8 µM), and 24 hr later, ACR was added to the culture medium at a dose of 6 mM. After 24 hr, cell viability was tested using the MTT test. As Figure 2 shows, telmisartan with concentrations of 0.025 and 0.0125 µM significantly increases the survival of cells compared with the ACR group (*P*<0.001). 

***In vivo***

***Effect of telmisartan on ACR-induced motor deficit***

**Figure 3:**
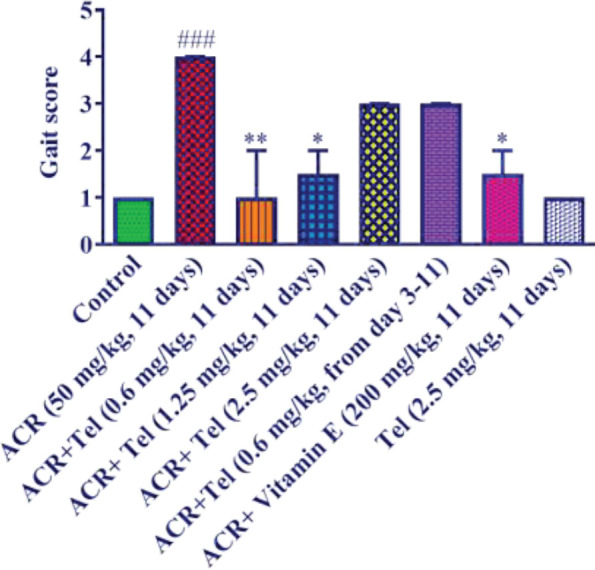
Effect of ACR (50 mg/kg), telmisartan (0.6, 1.25, and 2.5 mg/kg), and vitamin E (200 mg/kg) on the behavioral index (gait scores) in rats. The data are presented in mean±SD (n=6). The Nonparametric Kruskal-Wallis test was used for statistical analysis. ### *P*<0.001 compared with the control group, ** *P*<0.01 and * *P*<0.05 compared with the ACR group ACR: acrylamide; Tel: telmisartan

Intraperitoneal injection of ACR 50 mg/kg for 11 days caused clear movement disorders compared with the control group (*P*<0.001). Administering telmisartan at doses of 0.6 mg/kg (*P*<0.01) and 1.25 mg/kg (*P*<0.05) along with ACR could significantly reduce movement disorders compared with the ACR group. But telmisartan at the dose of 2.5 mg/kg along with ACR could not reduce movement disorder compared with the ACR group. In the treatment group (telmisartan 0.6 mg/kg, from day 3), there was no improvement in movement disorder in comparison with the ACR group. Moreover, vitamin E along with ACR could significantly reduce movement disorder versus the ACR group (*P*<0.05). Administering telmisartan at the dose of 2.5 mg/kg to healthy animals did not cause significant changes compared with the control group (Figure 3). 

***Effect of telmisartan on lipid peroxidation in brain tissue***

**Figure 4:**
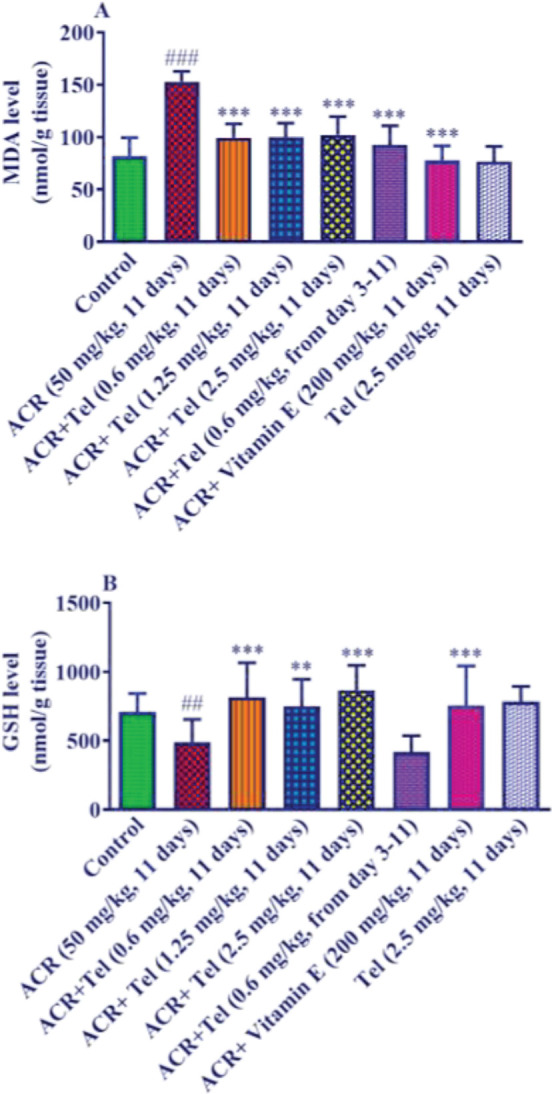
Effect of ACR (50 mg/kg), telmisartan (0.6, 1.25, and 2.5 mg/kg), and vitamin E (200 mg/kg) on A: MDA level and B: GSH level of the cerebral cortex of the brain. The data are presented as mean±SD (n=6). ANOVA and post-test Tukey–Kramer were used for statistical analysis. ### *P*<0.001 and ## *P*<0.01 compared with the control group, *** *P*<0.001 and ** *P*<0.01 compared with the ACR group

ACR: acrylamide; Tel: telmisartan

ACR injection (50 mg/kg, 11 days) caused a significant increase in the amount of MDA in the brain tissue compared with the control group (*P*<0.001). The doses of 0.6 mg/kg, 1.25 mg/kg, and 2.5 mg/kg of telmisartan along with ACR could significantly reduce MDA amount in the brain tissue compared with the ACR group (*P*<0.001). Also, the treatment group (telmisartan 0.6 mg/kg, from day 3 to 11) could significantly decrease MDA levels compared with the ACR group (*P*<0.001). Administration of vitamin E intraperitoneally for 11 days along with ACR caused a significant decline in MDA compared with the ACR group (*P*<0.001) (Figure 4A). Furthermore, no significant difference was seen in the group receiving telmisartan 2.5 mg/kg alone with the control group.

***Effect of telmisartan on GSH level in brain tissue***

Injection of ACR at the dose of 50 mg/kg for 11 days caused a significant decrease in the GSH content of brain tissue compared with the control group (*P*<0.01). Telmisartan at doses of 0.6 mg/kg (*P*<0.001), 1.25 mg/kg (*P*<0.01), and 2.5 mg/kg (*P*<0.001) along with ACR caused a significant increase in the content of GSH in the brain tissue compared with the ACR group. However, in the telmisartan treatment group (0.6 mg/kg, from day 3) along with ACR, there was no significant change in GSH content compared with the ACR group (Figure 4B). Vitamin E administration along with ACR resulted in a significant increase in GSH level compared with the ACR group (*P*<0.001). Also, no significant difference was observed in the group receiving 2.5 mg/kg telmisartan alone with the control group. 

***Effect of telmisartan on blood pressure***

**Figure 5:**
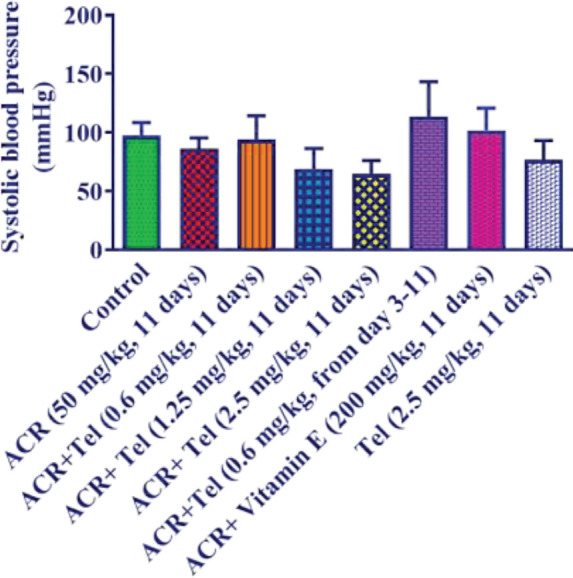
Effect of ACR (50 mg/kg), telmisartan (0.6, 1.25, and 2.5 mg/kg), and vitamin E (200 mg/kg) on systolic blood pressure. The data are presented as mean±SD (n=6). ANOVA and post-test Tukey–Kramer were used for statistical analysis

ACR: acrylamide; Tel: telmisartan

Administration of ACR, telmisartan, and vitamin E did not have a significant effect on the blood pressure of animals (Figure 5).

ACR: acrylamide, Tel: telmisartan

## Discussion

In the current study, the neuroprotective effects of telmisartan against the neurotoxicity caused by ACR in PC12 cells and rats were evaluated. The obtained data from the *in vitro* part of the study indicated that ACR 6 mM reduced the viability of PC12 cells versus the control group. However, pretreatment of these cells with telmisartan (0.0125 and 0.025 µM) could significantly increase the cell viability in comparison with the cells exposed to ACR. The findings of the *in vivo* part of the current work illustrated that the intraperitoneal injection of ACR into rats induced significant motor impairment. It also resulted in enhanced MDA amount and decreased GSH levels in brain cortical tissue. Compared with the ACR group, telmisartan (0.6, 1.25 mg/kg, 11 days) and vitamin E (200 mg/kg, every other day) significantly reduced locomotor abnormalities. In comparison with the ACR group, administration of telmisartan pre-treatment groups (0.6, 1.25, and 2.5 mg/kg, 11 days) and vitamin E lowered MDA levels and increased GSH content. Administration of telmisartan in the treatment group (most effective dose of 0.6 mg/kg from day 3–11 of ACR injection) could just increase the MDA level compared with the ACR group. But, it was not successful in ameliorating ACR-induced locomotor impairment and reducing GSH levels in cortical tissue. 

***In vitro***

The determined IC_50_ of ACR in this study was 6 mM, compared with reported values of 4.8 or 5.5 and 10 mM in previous papers (2, 12). This variation might be brought on by the variety of ACR purity and potency. Pre-treating PC12 cells with telmisartan 0.0125 and 0.025 µM could significantly enhance cell viability compared with the ACR group. Telmisartan at greater concentrations (> 0.05 µM) did not improve cell viability. This incident might be explained by this compound’s pro-oxidant activity at larger concentrations. In line with our results, it has been disclosed that pretreating neural stem cells with telmisartan (0.01, 0.1, 1, and 10 µM) enhanced the cell viability of these cells exposed to oxygen-glucose deprivation, but higher concentrations exhibited cytotoxic effects (25). Another research also claimed that pre-treating primary rat cerebellar granule cells exposed to nutrient deprivation-induced apoptosis with telmisartan 1 μM increased cell viability (26).

***In vivo***

ACR is a typical food toxin that is mostly produced in high-carbohydrate dishes that are deep-fried and baked (8). ACR has a neurotoxic impact on the neurological system in both animals and humans, which may result in several illnesses, such as neurotoxic syndrome, which has particular symptoms including ataxia, weight loss, and skeletal muscle weakness (27). Additionally, it has been demonstrated that ACR treatment degenerates neurons and destructs cellular macromolecules which are the main contributors to motor dysfunction (28). Likewise, in this study, rats that received intraperitoneal injections of ACR (50 mg/kg) for 11 days showed complete limb paralysis and began dragging their hind limbs. These ACR results were consistent with another study that found that after 11 days of exposure to ACR, the animals had developed paralysis (2, 29). In contrast, rats that received telmisartan in pre-treatment groups had less paralysis in their limbs. In line with our obtained data, another study reported that administration of telmisartan (10 mg/kg, 14 days, p.o.) to rats with Huntington’s disease resulted in noticeable amelioration in motor functions and muscle strength (15). Assessing the neuroprotective effect of oral administration of telmisartan (5 mg/kg/day, 3 weeks) in cuprizone-induced demyelination in mice revealed that telmisartan could significantly increase motor coordination and improve locomotor activity (14). According to our findings, although telmisartan (0.6 mg/kg) in the treatment group from day 3 to 11 decreased motor impairment, the reduction was not statistically significant. As a result, it is possible to assume that telmisartan requires more time than 8 days to significantly alleviate ACR-induced motor dysfunction. It is also crucial to note that the doses of telmisartan were chosen so as not to cause a decrease in the blood pressure of the animals. Because it was possible that the decrease in blood pressure would affect the animals’ movement activities and cause errors in data interpretation.

Moreover, ACR exposure can decrease GSH levels, increase MDA levels, and hurt brain tissue, demonstrating the critical role that oxidative stress plays in ACR-induced neurotoxicity (12, 16). In our investigation, the administration of ACR also led to an increase in MDA levels and a decrease in GSH levels in the brain tissue. Administration of telmisartan in both protocols (pretreatment and treatment groups) could decrease the MDA level compared with the ACR group. But, just pretreatment groups enhanced GSH content in cortical tissue in comparison with the ACR group. It has been indicated that administration of telmisartan (0.1 and 0.5 mg/kg, 7 days, IP) to mice with lipopolysaccharide-induced cognitive impairment decreased brain MDA and increased GSH amount (30). Likewise, telmisartan lessened the oxidative stress induced by 3-nitropropionic acid in rats (Huntington’s disease model) by augmenting the GSH level and decreasing the MDA level in brain tissue (15). 

Because vitamin E has been demonstrated to have a neuroprotective effect by acting as an anti-oxidant against ACR-induced neurotoxicity (2), our team administered it as a positive control in the current investigation. The current study found no difference between telmisartan in pre-treatment groups and vitamin E in terms of preventing oxidative stress in brain tissue. 

## Conclusion

Our findings demonstrate that oxidative stress is the origin of ACR neurotoxicity. In the cerebral cortex, telmisartan increases GSH levels and lowers lipid peroxidation. The anti-oxidant effect of telmisartan is regarded as the basic defense against ACR-induced toxicity. However, according to the obtained results, other properties of telmisartan such as anti-inflammatory, anti-apoptotic, and autophagy-modulating, may also play a role in causing the observed effects.

## Authors’ Contributions

H H and BM R supervised the whole project, conceived the original idea, verified the analytical methods, and checked the whole procedure and paper. Z Y performed the experiments and analyzed the data. MGR helped in doing the experiments, analyzing the data, and writing the manuscript. All authors have read and approved the paper.

## Conflicts of Interest

We declare that we have no conflicts of interest.
